# Lumbar Canal Stenosis Caused by Marked Bone Overgrowth after Decompression Surgery

**DOI:** 10.1155/2022/9462399

**Published:** 2022-06-03

**Authors:** Hiroya Shimauchi-Ohtaki, Manabu Minami, Toshiyuki Takahashi, Ryo Kanematsu, Fumiaki Honda, Junya Hanakita

**Affiliations:** ^1^Spinal Disorders Center, Fujieda Heisei Memorial Hospital, Fujieda, Shizuoka, Japan; ^2^Department of Neurosurgery, Gunma University Graduate School of Medicine, Japan

## Abstract

Narrowing of the lumbar canal due to bone regrowth after lumbar decompression surgery generally occurs at the facet joint; it is exceedingly rare for this phenomenon to occur at the laminar arch. Herein, we describe a case of restenosis caused by marked bone overgrowth at the facet joints and laminar arch after lumbar decompression surgery. A 64-year-old man underwent partial hemilaminectomy for lumbar canal stenosis at the L3/L4 level 12 years ago. His symptoms recurred 7 years after the first surgery. Overgrowth of the laminar arch and facet joints was observed at the decompression site. Thus, partial laminectomy of L3 and L4 was performed as a second surgery. Four years after the second surgery, a laminectomy of L3-L4 was performed for bone restenosis and disc herniation. The underlying mechanism of the remarkable overgrowth of the removed lamina remains unclear. Endochondral ossification signals and mechanosignals should be comprehensively examined.

## 1. Introduction

Bone regrowth after lumbar decompression surgery has been reported in the literature [1–4]. Specifically, bone regrowth has been noted in several radiographic imaging studies after several operative procedures [1–4]. However, most cases of bone regrowth after lumbar decompression surgery have not resulted in neurological symptoms. Particularly, nerve compression due to laminar arch overgrowth at the site of decompression is rare. In this report, we describe an extremely rare case of restenosis due to marked bone overgrowth at the facet joints and laminar arch following lumbar decompression surgery.

## 2. Case Presentation

**Figure 1:**
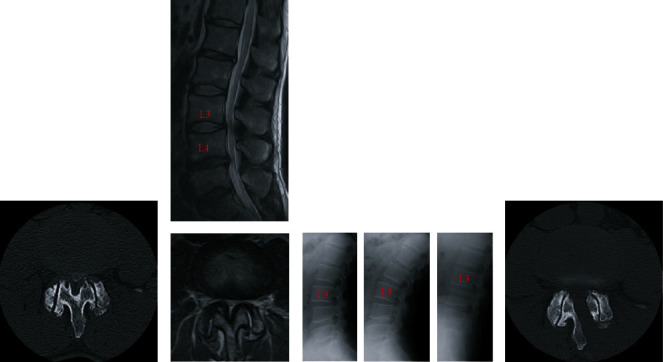
Radiographic images before and after the first surgery. (a) Computed tomography (CT) myelogram before the first surgery showing central canal stenosis at L3/L4. (b) Magnetic resonance (MR) image before the first surgery showing central canal stenosis and facet effusions at L3/4. (c) Dynamic X-ray images (neutral, extension, and flexion position) before the first surgery showing mild L3 spondylolisthesis with a 4-mm slip change. (d) CT image 5 days after the first surgery showing unilateral partial laminectomy at L3/L4.

**Figure 2:**
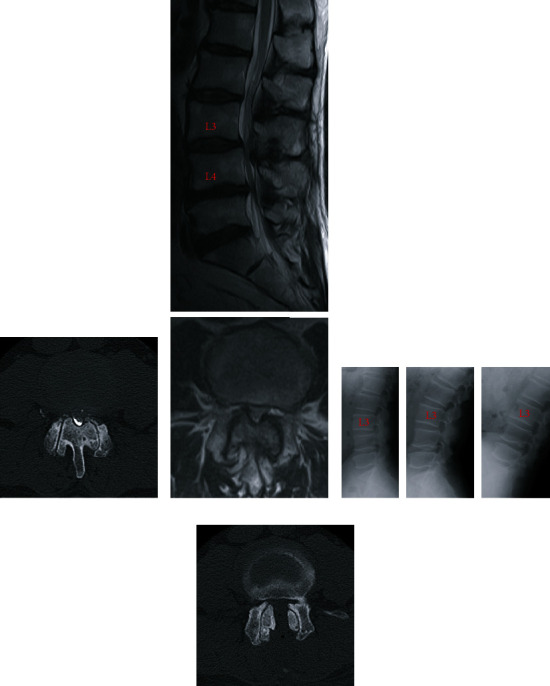
Radiographic images before and after the second surgery. (a) Computed tomography (CT) myelogram before the second surgery showing restenosis due to marked bone overgrowth of the lamina and facet joints. (b) Magnetic resonance (MR) image before the second surgery showing lumbar canal stenosis at L3/L4. (c) Dynamic X-ray images (neutral, extension, and flexion position) before the second surgery showing no significant changes in lumbar alignment or spinal stability. (d) CT image 4 days after the second surgery showing the results of the bilateral laminectomy at L3/L4.

**Figure 3:**
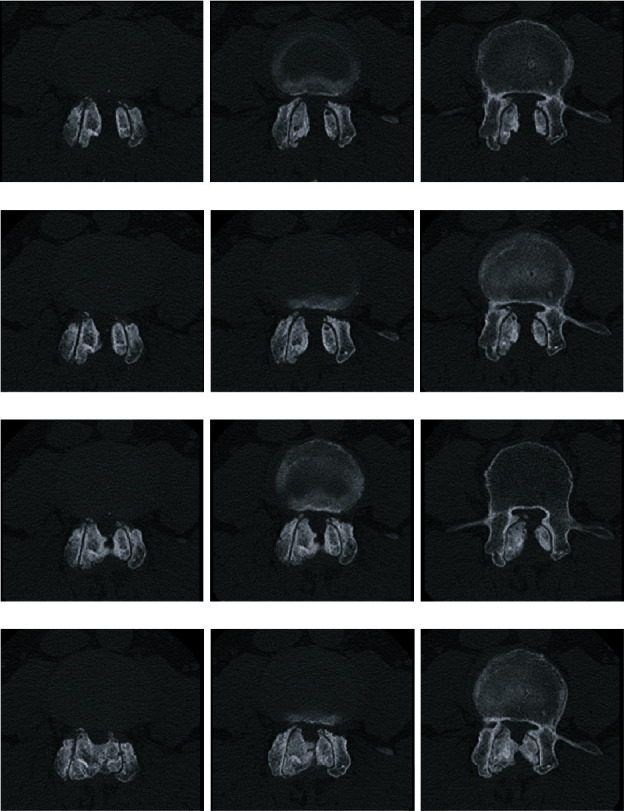
Computed tomography (CT) images after the second decompression surgery. (a) CT images 6 months after the second surgery. (b) CT images 1 year after the second surgery. (c) CT images 2 years after the second surgery showing bone regrowth mainly around the facet joints. (d) CT images 4.3 years after the second surgery showing the marked bone regrowth over the lamina.

**Figure 4:**
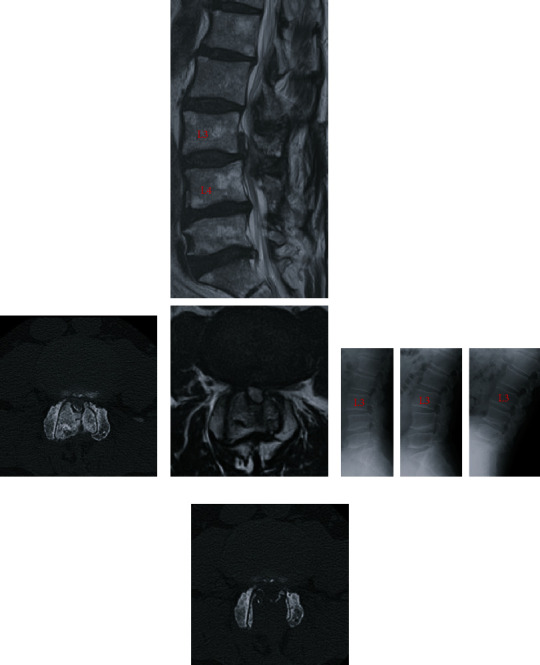
Radiographic images before and after the third surgery. (a) Computed tomography (CT) myelogram before the third surgery showing re-restenosis due to marked bone overgrowth of the lamina and facet joints. (b) Magnetic resonance (MR) image before the third surgery showing central canal restenosis due to marked bone overgrowth and disc herniation of L3/L4. (c) Dynamic X-ray images (neutral, extension, and flexion position) before the third surgery showing no significant changes in lumbar alignment or spinal stability. (d) CT image 1 day after the third surgery showing the results of the bilateral laminectomy at L3/L4.

A 64-year-old man experienced bilateral leg numbness and took limaprost alfadex, a prostaglandin E1 analog, for lumbar canal stenosis at the L3/L4 level. However, the symptoms in his legs were not improved by medical treatment. He underwent microscopic, unilateral, partial hemilaminectomy with bilateral flavectomy 12 years ago (“first surgery”; Figure 1). Before the first surgery, mild L3 spondylolisthesis with a 4-mm slip change was observed on dynamic X-ray examination (Figure 1(c)), and a facet effusion was detected by magnetic resonance imaging (Figure 1(b)). Although the neurological symptoms in his legs improved and the modified Japanese Orthopaedic Association score increased from 15/23 to 20/23 1 year after the first surgery, the patient reported a recurrence of bilateral leg pain and intermittent claudication 7 years after the first surgery. Bone over-regrowth of the laminar arch and facet joints was detected at the decompression site, by computed tomography (CT), which induced lumbar canal restenosis at the L3/L4 level (Figure 2(a)). Dynamic lumbar X-ray examination did not show overt progression of spondylolisthesis at the L3/4 level (Figure 2(c)). Prostaglandin E1 analog was ineffective in treating his symptoms; subsequently, a laminectomy of L3 and L4 was performed (“second surgery”; Figure 2(d)). The patient's symptoms improved again, and the visual analog scale score for lower limb and buttock pain was improved from 7/10 cm to 0/10 cm 1 year after the second surgery. Four years after the second surgery, the patient's bilateral leg pain recurred. CT and CT myelogram revealed canal stenosis due to marked bone overgrowth and disc herniation of L3/L4 (Figures 3 and 4(a)). No significant changes in lumbar alignment or spinal stability were observed (Figure 4(c)). A laminectomy of L3 and L4 was performed (“third surgery”; Figure 4(d)), and the patient's symptoms improved again following the surgery, and the visual analog scale score for lower limb and buttock pain was improved from 8/10 cm to 1/10 cm 6 months after the third surgery. The patient was tested for calcium metabolism and growth hormone disorders; none was detected. Pathological examination of the resected bone revealed hyperchondroplasia and endochondral ossification; no evidence of malignancy was found.

This study was approved by the Ethics Committee of the Fujieda Heisei Memorial Hospital, and the involved patient provided consent.

## 3. Discussion

In this case report, we describe an extremely rare case of lumbar canal stenosis with neurological symptoms due to marked bone overgrowth after lumbar decompression surgery. Bone regrowth has been known to occur at decompression sites [1–4]. Postacchini and Cinotti [4] reported that 88% of patients show bone regrowth 8.6 years after total or bilateral laminectomy, with two reported cases showing marked bone regrowth and lumbar canal restenosis. They reported that restenosis occurred at the posterior facet joints and that the regrowth of the lamina does not usually cause significant compression, except in degenerative spondylolisthesis [4]. Similarly, Chen et al. [1] reported that 94% of patients exhibit bone regrowth 4.5 years after total or partial laminectomy, and Guigui et al. [3] reported that most patients showed bone regrowth 8 years after total laminectomy, although it was mild. Spondylolisthesis [4] and postoperative instability [3] have been reported to be associated with bone regrowth. In addition, total block on myelogram, a longer follow-up period, multiple decompression levels, and younger age were also associated with bone regrowth [1].

In recent years, lumbar surgery has become less invasive as surgical procedures have advanced. Microscopic bilateral decompression via a unilateral approach is a less invasive surgical procedure compared with conventional laminectomy, which may help prevent postoperative spinal instability and marked bone regrowth [2]. However, in the present case, marked postoperative bone regrowth induced lumbar canal restenosis not only at the facet joints but also at the lamina, despite the fact that the first surgery was less invasive than the second surgery. Notably, no significant changes in lumbar stability or alignment were found between the three preoperative dynamic X-ray images (Figures 1(c), 2(c), and 4(c)). Micromotion could induce the excessive hypertrophy of facet joints. CT images over time suggested that it could also lead to the hypertrophy of the laminar arch continuing from the facet joints (Figure 2). In order to treat restenosis, the patient had repeated decompression surgeries for both the second and the third surgery on his request, although we considered other surgical methods including fusion surgery for the third surgery. It was reported that spinal stabilization could decrease the thickness of the ossification of the posterior longitudinal ligament and protruded bone fragments in the spinal canal of burst fractures [5, 6]. Therefore, fusion surgery could prevent the need for reoperation of the excessive bone restenosis of facet joints and the laminar arch with micromotion of vertebra, such as in the present case. Notably, excessive bone regeneration was more remarkable after the decompression surgery. Although degenerated medial hypertrophic facet changes participate in the central canal stenosis [7], hypertrophy of the laminar arch continuing from the facet joints is rare during the natural course. Therefore, bone regeneration after decompression surgery could surpass the natural course of bone hypertrophy, and it could be an essential factor to consider in deciding which reoperative method to use.

In the present case, hyperchondroplasia and endochondral ossification were observed on pathological examination. Endochondral or intramembranous ossification is required for bone regrowth, and various signal transduction pathways—such as Hedgehog, Notch, Wnt, bone morphogenetic protein signaling, and fibroblast growth factor signaling—that work through transcriptional regulation have been reported [8]. It is possible that some signaling abnormalities may be detected not only in known disease processes but also in the excessive postoperative bone regrowth, as in the present case. Interestingly, systemic abnormalities were not apparent in this case, and the excessive postoperative bone overgrowth consistently occurred at the same decompression site. Although mechanosignaling abnormalities may induce such excessive bone regrowth, the underlying mechanism of the remarkable laminar overgrowth in this case remains unclear. Thus, endochondral ossification signals and mechanosignals should be comprehensively examined as potential causes of bone overgrowth, and may provide new avenues for osseous lumbar canal stenosis and postoperative bone overgrowth research.

## 4. Conclusion

We describe an extremely rare case of lumbar canal stenosis with neurological symptoms due to marked bone overgrowth after lumbar decompression surgery. The underlying mechanism of the remarkable laminar overgrowth in this case remains unclear. Endochondral ossification signals and mechanosignals should be comprehensively examined as potential causes of bone overgrowth.
